# Kinetically Stabilizing Mutations in Beta Tubulins Create Isotype-Specific Brain Malformations

**DOI:** 10.3389/fcell.2021.765992

**Published:** 2021-11-18

**Authors:** Kristen Park, Katelyn J. Hoff, Linnea Wethekam, Nicholas Stence, Margarita Saenz, Jeffrey K. Moore

**Affiliations:** ^1^ Department of Pediatrics and Neurology, Children’s Hospital Colorado, University of Colorado Anschutz Medical Campus, Aurora, CO, United States; ^2^ Department of Cell and Developmental Biology, University of Colorado Anschutz Medical Campus, Aurora, CO, United States; ^3^ Section of Pediatric Radiology, Department of Radiology, Children’s Hospital Colorado, University of Colorado Anschutz Medical Campus, Aurora, CO, United States; ^4^ Section of Genetics, Department of Pediatrics, Children’s Hospital Colorado, University of Colorado Anschutz Medical Campus, Aurora, CO, United States

**Keywords:** cytoskeleton, microtubule, tubulin, tubulinopathy, brain development

## Abstract

Mutations in the family of genes encoding the tubulin subunits of microtubules are associated with a spectrum of human brain malformations known as tubulinopathies. How these mutations impact tubulin activity to give rise to distinct developmental consequences is poorly understood. Here we report two patients exhibiting brain malformations characteristic of tubulinopathies and heterozygous T178M missense mutations in different β-tubulin genes, *TUBB2A* or *TUBB3*. RNAseq analysis indicates that both *TUBB2A* and *TUBB3* are expressed in the brain during development, but only *TUBB2A* maintains high expression in neurons into adulthood. The T178 residue is highly conserved in β-tubulins and located in the exchangeable GTP-binding pocket of β-tubulin. To determine the impact of T178M on β-tubulin function we created an analogous mutation in the β-tubulin of budding yeast and show that the substitution acts dominantly to produce kinetically stabilized microtubules that assemble and disassemble slowly, with fewer transitions between these states. *In vitro* experiments with purified mutant tubulin demonstrate that T178M decreases the intrinsic assembly activity of β-tubulin and forms microtubules that rarely transition to disassembly. We provide evidence that the T178M substitution disrupts GTPase-dependent conformational changes in tubulin, providing a mechanistic explanation for kinetic stabilization. Our findings demonstrate the importance of tubulin’s GTPase activity during brain development, and indicate that tubulin isotypes play different, important roles during brain development.

## 1 Introduction

Tubulin is the protein building block of the microtubule cytoskeleton and accounts for 25% of total protein in the mammalian brain, by far the most abundant GTPase in the brain (Hiller and Weber, 1978). GTPase activity is essential for tubulin function, particularly during brain development when dynamic microtubules support billions of cell divisions and the subsequent migration and process extension events that generate the complex architecture of the human adult brain. Each tubulin heterodimer binds to two molecules of GTP. One GTP binds to a site buried within tubulin and hydrolyzes very slowly (Weisenberg et al., 1968; Spiegelman et al., 1977). The second GTP binds to the so-called exchangeable site on β-tubulin, is hydrolyzed during assembly into microtubules and exchanges when tubulin is in solution (Kobayashi, 1975; Nogales et al., 1998). GTPase activity at the exchangeable site controls a switch in tubulin conformation that creates a six-fold greater microtubule association for the GTP-bound state (Carlier and Pantaloni, 1978). These differences in assembly kinetics drive dynamic instability, the ability of microtubules to stochastically transition between phases of growth and shortening, that permit rapid generation and remodeling of the microtubule cytoskeleton.

The tubulin heterodimer consists of α- and β-tubulin subunits, which are each encoded by families of genes known as tubulin isotypes. The human genome encodes 9 α-tubulin isotypes and 8–10 β-tubulin isotypes (Findeisen et al., 2014). In general, tubulin isotypes exhibit unique expression programs that differ across cell types and across development (Luduena and Banerjee, 2008). Because of these unique expression programs, tubulin isotypes are hypothesized to play distinct roles throughout human development, particularly during the complicated process of neurodevelopment. In support of this hypothesis, mouse models harboring null mutations in the α-tubulin isotype *Tuba1a* exhibit severe defects in brain development, while null mutations in the β-tubulin isotypes *Tubb2a* and *Tubb2b* exhibit milder cortical malformations (Bittermann et al., 2019). In contrast, a separate study examined mice with null mutations in the β-tubulin isotype, *Tubb3*, which is expressed in neurons during brain development (Burgoyne et al., 1988; Chen et al., 2003; Katsetos et al., 2003), and reported no clear brain development phenotypes (Latremoliere et al., 2018). This suggests that loss of function mutations in some isotypes may be compensated for by upregulation of alternative isotypes. The roles of isotypes in metazoan development remains a frontier in the microtubule field.

Over the past two decades, mutations impacting the genes encoding tubulins have emerged as major genetic causes of brain development disorders. These include ‘tubulinopathies’ which are characterized as cortical malformations linked to *de novo*, heterozygous missense mutations in tubulin isotypes (Keays et al., 2007; Poirier et al., 2010; Bahi-Buisson et al., 2014; Cushion et al., 2014; Fukumura et al., 2016; Mutch et al., 2016; Aiken et al., 2017; Di Donato et al., 2017; Ejaz et al., 2017; Rodan et al., 2017; Severino et al., 2020; Brock et al., 2021). In addition, cases of the postnatal brain development disorder hypomyelination with atrophy of the basal ganglia and cerebellum (H-ABC) are linked to heterozygous missense mutations in the β-tubulin isotype *TUBB4A* (Hersheson et al., 2013; Lohmann et al., 2013; Simons et al., 2013; Hamilton et al., 2014; Miyatake et al., 2014; Purnell et al., 2014; Lu et al., 2017). As the spectrum of phenotypes and associated mutations continues to increase, two major questions remain unresolved: 1) how do the different ⍺- and β-tubulin isotypes contribute to normal brain development such that mutations in different genes might lead to unique developmental consequences; and 2) what are the mechanisms through which patient-associated missense mutations alter tubulin protein function to lead to aberrant development?

This study seeks to address both questions. We describe two patients with analogous T178M mutations in either the *TUBB2A* or *TUBB3* β-tubulin isotypes exhibiting cortical malformations consistent with tubulinopathies. Our analysis of RNA expression is consistent with roles for *TUBB2A* and *TUBB3* during brain development, particularly in neurons. In contrast, a previous report identified a T178M mutation in the *TUBB4A* isotype of a patient exhibiting H-ABC (Tonduti et al., 2016). Our analysis indicates that *TUBB4A* expression is primarily limited to oligodendrocytes and increases during postnatal brain development. To investigate the mechanistic impact of T178M on tubulin protein activity, we modeled the mutation in yeast β-tubulin. Our results show that T178M weakens the assembly activity of purified tubulin protein, but when expressed as a heterozygous mutation in cells promotes the formation of stable microtubules with diminished dynamic instability. We conclude that T178M disrupts the control of dynamic instability by tubulin’s GTPase to create kinetically stabilized microtubules. When present in different β-tubulin isotypes, this mutation dominantly alters microtubule activity in cells that express the affected isotype.

## 2 Results

### 2.1 Clinical History of TUBB2A and TUBB3 Patients

#### 2.1.1 Patient 1

**FIGURE 1:**
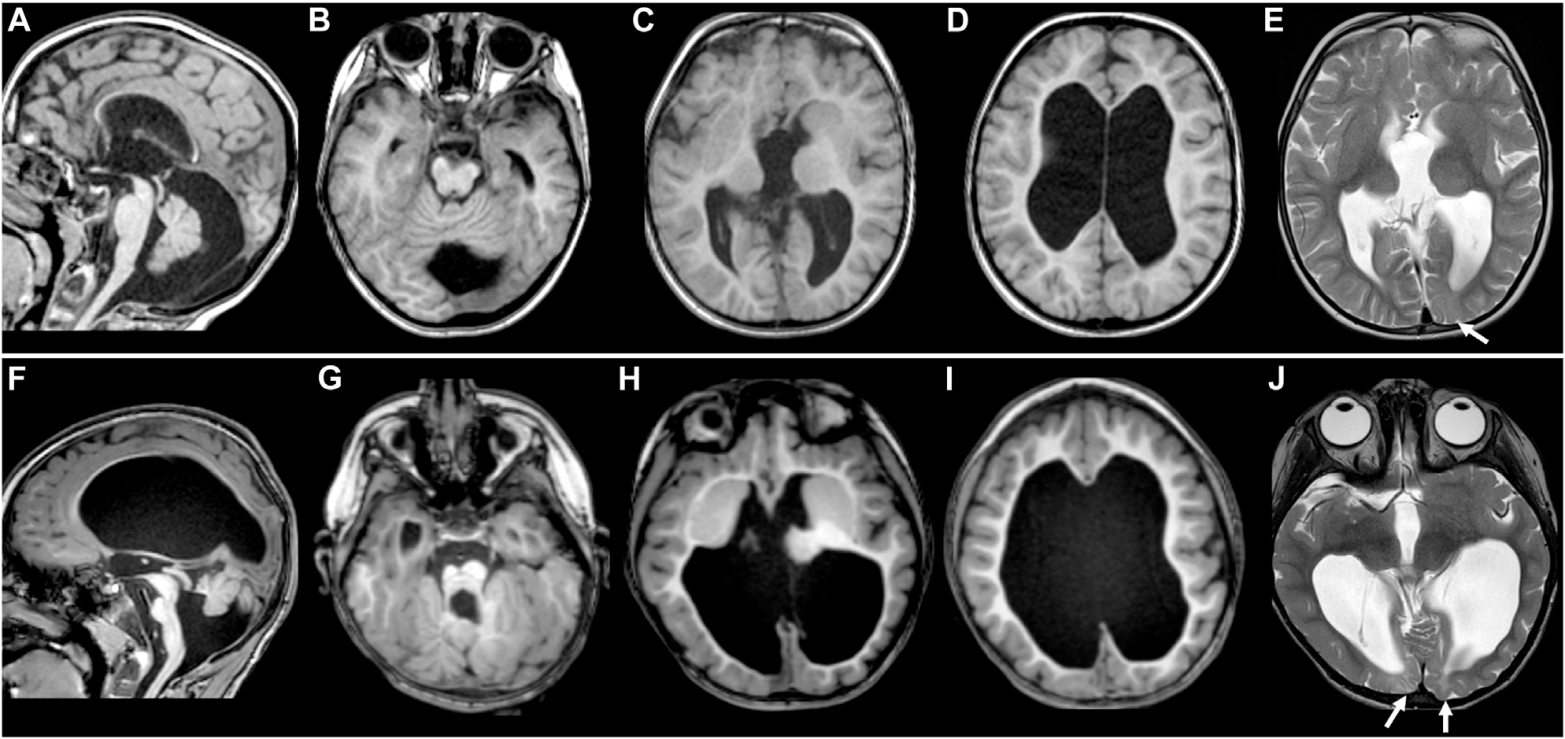
MR images of *TUBB2A*-T178M and *TUBB3*-T178M patients. Selected axial T1 **(A–D)** and T2 **(E)** MR images of patient #1 (with *TUBB2A* mutation) obtained at age 4 demonstrate common features of a moderate tubulinopathy MR phenotype, including **(A)** mildly small pons and vermis, thin corpus callosum; **(B)** small, asymmetric-appearing brainstem, mild superior vermian disorganization; **(C)** abnormal, asymmetric basal ganglia with enlarged caudate and thin anterior limb internal capsule separating the caudate and putamen; **(C,D)** diffuse pattern of tubulinopathy-associated dysgyria consisting of normal-thickness but closely spaced and disorganized-appearing sulci with orientation directed radially to a central point; **(D)** ventriculomegaly with low white matter volume. **(E)** also demonstrates asymmetric T2 hyperintensity in the juxtacortical white matter of the left occipital lobe (arrow), with preservation of overlying cortex. Selected axial T1 (**F–I** and T2 **(J)** MR images of patient #2 (with *TUBB3* mutation) obtained at age 3 demonstrate similar features as patient #1, but of a more severe MRI phenotype, including **(F)** moderately to markedly small pons and vermis with posterior vermian rotation, mildly thickened tectum relative to brainstem size, thin corpus callosum; **(G)** small, asymmetric-appearing brainstem, moderate/severe superior vermian and cerebellar foliar disorganization; **(H)** abnormal, asymmetric basal ganglia with no discernable separation of the caudate head and putamen; **(H,I)** tubulinopathy-associated dysgyria with shallow, closely spaced and disorganized-appearing sulcation also directed radially to a central point; **(I)** ventriculomegaly with low white matter volume. **(J)** shows more extensive and intense abnormal T2 hyperintensity in the juxtacortical white matter of the bilateral occipital lobes (arrowheads), with preservation of overlying cortex.

The index patient was born via vaginal delivery at term to a 31-year-old G3P1 mother after an uncomplicated pregnancy. Birth weight was 8l bs 4 oz (50–75%), length was 22.5 inches (>97%), occipitofrontal circumference (OFC): 35 cm (25–50%) with normal Apgar scores. Developmental delays were noted early on when he did not roll or sit on time. Initial neurologic evaluation was performed due to concern for ventriculomegaly on head ultrasound at 9 months of age. Subsequent MRI revealed significant abnormalities, as described below (Figures 1A–E). He was noted on exam to have poor visual fixation and tracking such that Ophthalmology evaluation was recommended. This revealed cortical visual impairment, mild optic nerve hypoplasia, right exophoria, and strabismic amblyopia. He was referred to Neurology at 11 months of age due to emerging microcephaly (head circumference = 44 cm, 7%, *Z* = −1.5) in addition to the above concerns. Myoclonic seizures began at approximately 16 months of age and have not resolved with trials of two appropriate anticonvulsant medications. Most recent electroencephalogram (EEG) performed at 2 years 11 months showed diffuse slowing and attenuation along with an absence of a posterior dominant rhythm, generalized spikes, as well as multifocal independent spike discharges. He underwent a number of non-diagnostic genetic and metabolic evaluations with several providers culminating in trio whole exome sequencing at 4 years of age. This identified a heterozygous *de novo* c.533C>T (p.T178M) substitution in the *TUBB2A* gene. His most recent examination at 11 years of age noted dysmorphic facial features including widely spaced teeth, a pronounced maxilla, and bilateral epicanthal folds. From a developmental standpoint, he acquired head control at 6 months and sat at approximately 3 years of age. Now at age 11, he crawls for locomotion, can walk short distances with a walker, is working on feeding himself, and uses an assistive communication device to indicate his wants and needs as he is largely non-verbal. Family has never reported mirror movements (Nissenkorn et al., 2021) nor has this been indicated on reviewed neurologic examinations. Current seizure frequency is 2–3 myoclonic seizures per day on valproic acid monotherapy, which has been stable.

#### 2.1.2 Patient 2

Patient 2 was born at 38 weeks gestation by vaginal delivery to a 40-year-old G11P4-5 mother after a pregnancy complicated only by the diagnosis of fetal brain malformations on routine ultrasound, felt to be consistent with semilobar holoprosencephaly. Birth weight was 6 lbs 12 oz (10–25%), length was 20 inches (50–75%), and OFC: 30.5 cm (0.1%, *Z* = −4.4) with normal Apgar scores. Neonatal MRI performed on DOL1 showed several abnormalities, as described below (Figure 1F–J). Ophthalmology evaluation revealed intermittent R exotropia and hyperopia, dysconjugate gaze, and strabismus. Seizures began at 1–2 months and were initially myoclonic in nature but subsequently, infantile spasms and focal seizures emerged which have been refractory to multiple medications and the ketogenic diet (initiated at age 3 years). Chromosomal microarray (180K with 5-cell screen) was performed prior to discharge from the NICU and did not identify any copy number variations. She had a mildly elevated Creatine Kinase (CK) on serum from DOL1 (247, normal range 29–168 IU/L) such that a muscular dystrophy next generation sequencing (NGS) panel (sequencing of 33 genes and del/dup of 4; see *Materials and Methods*) was performed without significant findings (Mendell et al., 2012). This was followed by testing for congenital disorders of glycosylation which was also negative. She was referred to our neurogenetics clinic at 3 years of age where repeat review of her MRI was suggestive of a tubulinopathy such that targeted testing for cortical brain malformation genes via NGS was performed (56 in panel; see *Materials and Methods*) and identified a heterozygous pathogenic c.533C>T (p.T178M) substitution in *TUBB3*. Parental testing was not completed. At her last examination (6 years) she was non-verbal and non-ambulatory with mixed tone. She spends time in a stander for weight bearing. She is fed by gastrostomy tube only due to a combination of swallowing dysfunction and oral aversion. Both epileptic spasms and focal motor seizures occur on a daily basis despite combination therapy with vigabatrin and clobazam in addition to the ketogenic diet, although family does report improved alertness with the latter. Most recent EEG performed at 4 years of age showed a diffusely slow background with poor organization, slow spike and wave patterns, and multifocal epileptiform discharges.

### 2.2 Magnetic Resonance Imaging

Both patients demonstrated most of the typical imaging features previously described in tubulin gene mutations (Mutch et al., 2016; Di Donato et al., 2017; Severino et al., 2020; Brock et al., 2021), although the phenotype for patient #2 was generally more severe than that of patient #1. These findings included thin corpus callosum, dysgyria (sometimes referred to as a tubulinopathy-associated dysgyria) (Di Donato et al., 2017), small and disorganized cerebellar vermis, small and asymmetric-appearing brainstem, abnormal basal ganglia, and ventriculomegaly with low white matter volume (Figures 1A–D, F–I). While both patient’s white matter volumes were low, they were for the most part normally myelinated. However, small areas of white matter in the occipital lobes of both patients demonstrated abnormal T2 hyperintensity with preserved overlying cortex (Figures 1E,J; see arrows).

### 2.3 Expression Levels of β-Tubulin Isotypes During Neural Development

A previous study reported an analogous T178M mutation in the *TUBB4A* β-tubulin isotype, in a patient exhibiting hypomyelinating leukodystrophy (Tonduti et al., 2016). The different clinical features of that patient, compared to the patients described here motivated us to ask whether differences could be attributed to the expression of the *TUBB4A*, *TUBB3*, and *TUBB2A* β-tubulin isotypes throughout neurodevelopment.

**FIGURE 2:**
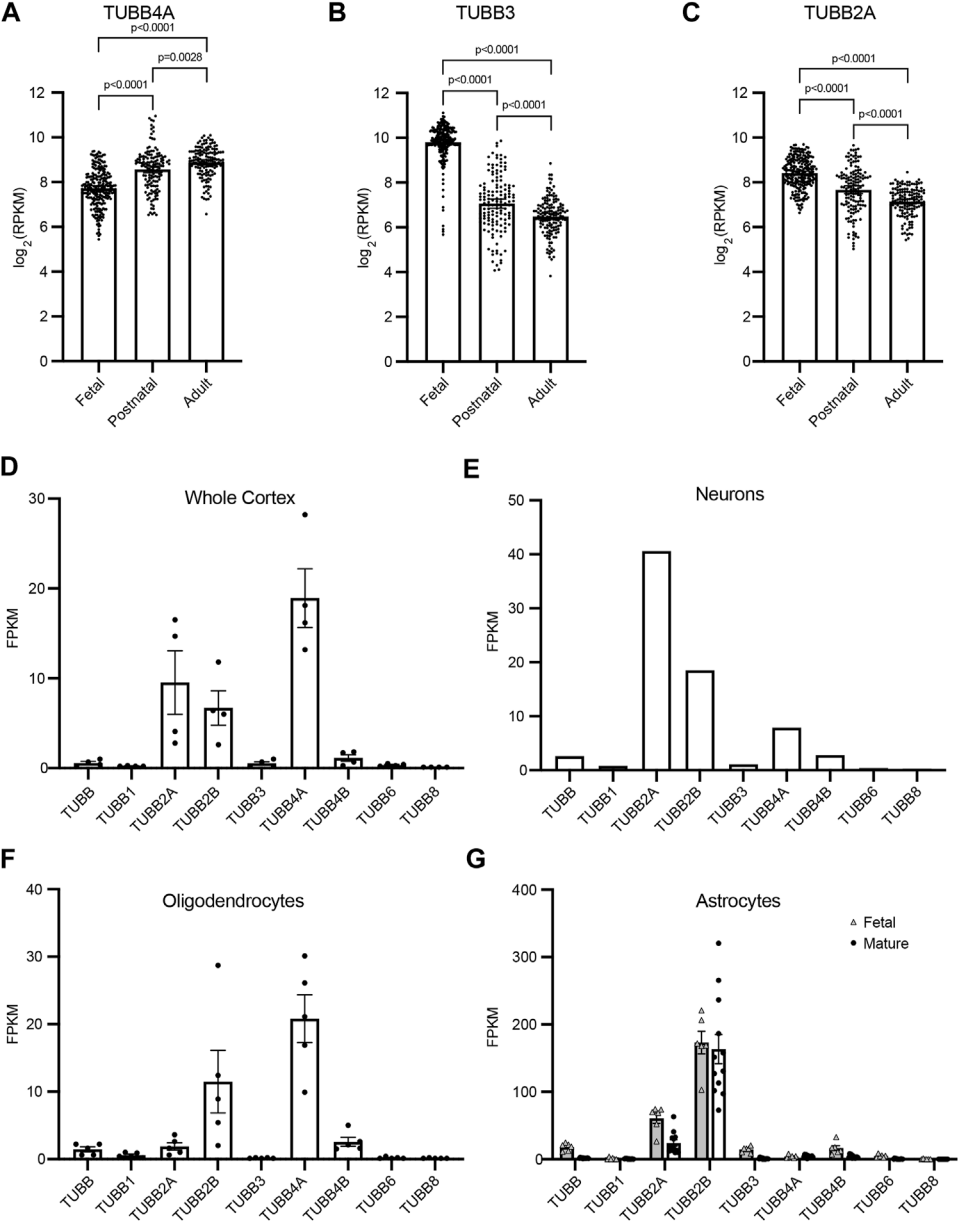
β-tubulin isotype composition is specific during developmental time periods and different cell types. **(A–C)** Expression of human β-tubulin isotype transcripts, *TUBB4A* **(A)**, *TUBB3* **(B)**, *TUBB2A* **(C)**, across development. Data obtained from cortical and subcortical samples publicly available RNA-sequencing data (Miller et al., 2014). Data binned according to developmental time point; fetal (8–37 pcw), postnatal (4 months–11 years), adult (13–40 years). Each data point represents data from an individual patient. Bars are the mean values and error bars represent the 95% confidence interval. *p*-values were determined by one-way ANOVA. **(D–G)** Transcript expression of human β-tubulin isotypes in the adult whole cortex **(D)**, neurons **(E)**, oligodendrocytes **(F)**, and fetal and mature astrocytes **(G)**. Data obtained from publicly available RNA-sequencing data (Zhang et al., 2016). Each data point represents data from an individual patient. Bars are the mean values and error bars represent the standard error of the mean.

The demand for tubulin varies throughout the course of neurodevelopment, and the expression of different tubulin isotypes changes to meet this demand. To gain insight into how *TUBB4A*, *TUBB3*, and *TUBB2A* expression temporally changes during development, we analyzed publicly available RNA-sequencing data from whole brain cortex samples (Miller et al., 2014). *TUBB4A* transcript levels increase significantly between the fetal and postnatal samples, followed by a smaller increase between postnatal and adult brains (Figure 2A). This is consistent with a role for *TUBB4A* in later stages of development, such as the myelination that occurs in postnatal brains (Miyatake et al., 2014; Tonduti et al., 2016; Joyal et al., 2019). In contrast, *TUBB3* transcript levels decrease approximately 30% between the fetal and postnatal brain samples (Figure 2B). *TUBB2A* transcript levels decrease steadily from fetal to postnatal to adult brains (Figure 2C). These data suggest an enhanced role for *TUBB3* and *TUBB2A* during early brain development.

Tubulin isotype expression varies not only across the timing of neurodevelopment, but also across different cell types. Therefore, we used publicly available RNA-sequencing data to compare transcript levels of nine β-tubulin isotypes in whole cortex samples and samples of isolated neurons, oligodendrocytes and astrocytes (Zhang et al., 2016). These data provide insight into both the blend of β isotypes in different cell types in the brain, as well as how the relative abundance of each isotype changes between cell types. *TUBB4A* transcript is abundant in the whole cortex sample and accounts for the largest portion of β isotypes in oligodendrocytes, but is diminished in neurons and astrocytes (Figures 2D–G). Patients with the T178M mutation in *TUBB4A* suffer from hypomyelination, which is in accordance with this isotype being dominantly expressed and important in oligodendrocytes. In contrast, *TUBB3* makes up a very small portion of the β-tubulin transcripts in the whole cortex and cell-type specific samples in this dataset (Figures 2D–G). However, these data (aside from the astrocytes) are obtained from adult tissue samples and would not reflect *TUBB3* transcript levels during fetal and postnatal neurodevelopment. *TUBB2A* transcript is expressed at moderate levels in samples from the whole adult cortex, but also shows cell-type specific enrichment. It dominates the β-tubulin pool in neurons, is scarcely expressed in oligodendrocytes, and middling in fetal and mature astrocytes (Figures 2D–G). Together, this RNA-sequencing data supports the hypothesis that the T178M mutation in the *TUBB3* and *TUBB2A* isotypes disrupt tubulin function during early neurodevelopment, and particularly in neurons, leading to changes in cortical architecture and brain size consistent with the clinical observations made in these patients.

### 2.4 T178M Decreases Tubulin Assembly Activity

**FIGURE 3:**
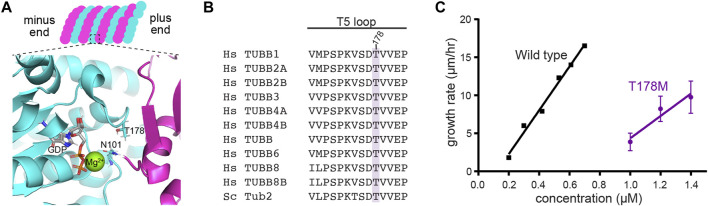
T178M impairs the assembly activity of β-tubulin. **(A)** Model of the GTP-bound state of the exchangeable site in tubulin, showing the position of T178 in the β-tubulin T5 loop and N101 in the T3 loop. The structure shown here is from porcine brain tubulin assembled in GTP and saturated in BeF_3_^−^ to mimic the GTP-bound state (pdb 6gze; Estevez-Gallego et al., 2020). **(B)** Alignment of the T5 loop from 10 human β-tubulin isotypes and the budding yeast β-tubulin Tub2. **(C)** Microtubule growth rates for indicated concentrations of wild-type and tub2-T178M mutant tubulin. Error bars represent SD.

We next examined how the T178M substitution impacts tubulin function. Threonine 178 is positioned within β-tubulin’s T5 loop, a highly conserved region that forms part of the exchangeable nucleotide-binding pocket between tubulin heterodimers (Figures 3A,B). Because the cycle of GTP binding, hydrolysis and GDP release at the exchangeable site plays a central role in regulating tubulin’s assembly activity, we tested how T178M affects tubulin assembly. We generated the analogous T178M mutation in the budding yeast β-tubulin gene *TUB2* and used an inducible expression system to purify yeast ⍺β-tubulin heterodimers containing tub2-T178M (Johnson et al., 2011). We measured assembly activity by incubating a range of concentrations of tub2-T178M tubulin heterodimers or wild-type control tubulin heterodimers in seeded assembly assays, and imaged microtubule dynamics by DIC microscopy (see Materials and Methods). We find that tub2-T178M tubulin assembles into microtubules; but only at concentrations ≥1 µM. In contrast, wild-type tubulin assembles at a five-fold lower concentration (Figure 3C). We determined the concentration dependent rate of microtubule assembly for tub2-T178M tubulin to be 14.7 µm/h/µM, compared to 28.7 µm/h/µM for wild-type tubulin (Figure 3C). Together, these data indicate that the T178M substitution in β-tubulin weakens the assembly activity of tubulin heterodimers.

While conducting these experiments, we found that microtubules assembled from tub2-T178M mutant tubulin exhibit fewer catastrophes—transitions from assembly or pause states to disassembly—than microtubules assembled from wild-type control tubulin. Whereas microtubules assembled from wild-type yeast tubulin exhibit an average of one catastrophe per 20 min of assembly time (Geyer et al., 2015), we observed no catastrophe events for all T178M mutant microtubules during 402 min of total observed assembly time.

### 2.5 T178M Dominantly Inhibits Microtubule Dynamics

Having established that the T178M weakens tubulin’s assembly activity, we next examined microtubule dynamics in cells, where tubulin activity is controlled by a wide variety of extrinsic regulatory proteins. We generated the T178M mutation at the chromosomal *TUB2* locus, the sole β-tubulin isotype in budding yeast. Heterozygous diploids expressing one copy of *tub2-*T178M and one copy of wild-type *TUB2* are viable; however, we were unable to recover haploid cells expressing *tub2-*T178M as the only source of β-tubulin. This result indicates that *tub2*-T178M alone is not sufficient to support β-tubulin function in yeast. Our characterization of T178M in yeast is therefore conducted in heterozygous diploids expressing one copy of *tub2*-T178M and one copy of wild-type *TUB2*.

**FIGURE 4:**
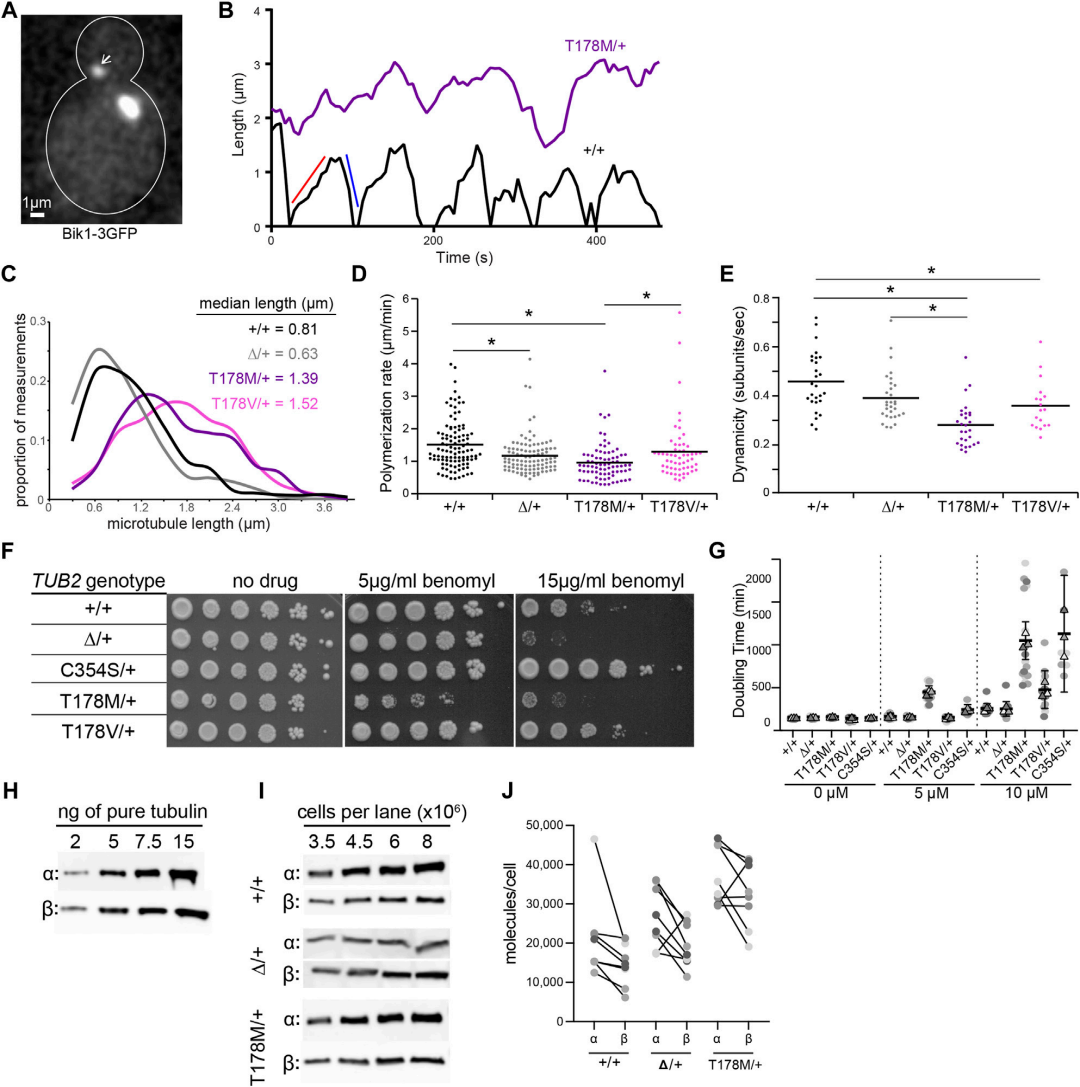
The β-tubulin T178M mutation dominantly decreases microtubule dynamics *in vivo*. **(A)** Representative image of microtubules in a cell labelled with Bik1-3GFP. Scale bar = 1 µm. **(B)** Representative life plots of astral microtubules in wild type, TUB2*/tub2Δ*, and TUB2*/tub2-T178M* cells. Astral microtubule length was measured over time by plotting the distance between plus-end-associated Bik1-3GFP and the proximal spindle pole. Red and blue lines on wild type plot show a single polymerization and depolymerization rate respectively. **(C)** Histogram of astral microtubule length measurements in pre-anaphase cells. +/+, *n* = 27 cells; ∆/+, *n* = 30 cells; T178M/+, *n* = 28 cells; T178V/+, *n* = 18 cells. **(D)** Polymerization rates of astral microtubules in pre-anaphase cells. Dots are individual polymerization events. Lines indicate the mean. Asterisks indicate statistical significance (alpha = 0.05) determined by Tukey-Kramer *post hoc* test. **(E)** Dynamicity of astral microtubules in pre-anaphase cells. Dots are values from individual cells, each imaged for 8 min. Lines indicate the mean. Asterisks indicate statistical significance (alpha = 0.05) determined by Tukey-Kramer *post hoc* test. **(F)** 10-fold dilution series of indicated strains were spotted to rich media (YPD; ‘‘no drug’’) or rich media supplemented with benomyl (5 or 15 µg/ml) and grown at 30°C. **(G)** Doubling time in minutes at 30°C of diploid yeast cells with indicated genotypes were calculated from change in absorbance (OD600) over time in the presence of indicated concentration nocodazole or DMSO-only control. Dots represent doubling time measurements for each technical replicate from three separate experiments, and the mean value of the technical replicates within each experiment is represented as a triangle. Bars indicate mean with 95% CI. **(H,I)** Western blots of dilution series of purified yeast α- and β-tubulin proteins and protein lysates from cells with indicated genotypes. **(J)** Estimated molecules/cell for wild type, *TUB2/tub2Δ*, and *TUB2/tub2-T178M* cells. Each pair of connected dots represents estimates for α- and β-tubulin from a technical replicate.

We measured microtubule dynamics by fusing three copies of GFP to the microtubule plus-end tracking protein Bik1/CLIP-170 at its native chromosomal locus (Figure 4A). We also attempted an alternative approach of ectopically expressing a fusion of GFP to ⍺-tubulin (GFP-Tub1); however, expressing this fusion severely impaired the viability of *tub2*-T178M heterozygotes (data not shown). Figure 4B shows representative “lifeplots” of individual astral microtubule lengths over time in a wild-type diploid cell and a *tub2-*T178M heterozygous cell expressing Bik1-3GFP. The complete set of microtubule dynamics parameters measured in this experiment is reported in Table 1. Our analysis reveals key differences for microtubule dynamics in *tub2-*T178M heterozygotes compared to wild-type controls: 1) astral microtubules are longer (Figure 4C), 2) exhibit slower polymerization rates (Figure 4D), and 3) exhibit an overall lower level of microtubule length change over time, known as “dynamicity” (Figure 4E). In general, microtubules in the tub2-T178M heterozygotes tend to persist in a “paused” state where they do not undergo sustained polymerization or depolymerization (Table 1).

**TABLE 1:** Dynamics of astral microtubules measured in diploid yeast cells during preanaphase.

Microtubule dynamics	+/+	+/∆	+/T178M	+/T178V	*p*-value from single factor ANOVA
Polymerization rate (µm/min)	1.51 ± 0.14	1.17 ± 0.11^a^	0.96 ± 0.13^a^	1.29 ± 0.24	2.20E-06
Depolymerization rate (µm/min)	2.28 ± 0.21	1.88 ± 0.16^a^	1.15 ± 0.12^a^	1.66 ± 0.24^a^	1.72E-14
Dynamicity (subunits/s)	0.46 ± 0.05	0.39 ± 0.04	0.28 ± 0.03^a^	0.34 ± 0.05^a^	1.35E-07
Catastrophe frequency (events/min of polymerization time)	0.92 ± 0.12	0.86 ± 0.11	0.78 ± 0.14	0.69 ± 0.11	0.07
Rescue frequency (events/min of depolymerization time)	1.43 ± 0.23	1.33 ± 0.24	0.99 ± 0.29	0.97 ± 0.28	0.03
% Time paused	20.8 ± 5.0	22.5 ± 5.8	35.6 ± 7.7^a^	31.4 ± 8.7	0.01
Number of cells analyzed	27	30	28	18	—

Image series were collected at 5 s intervals for 8 min and astral microtubule lengths were measured at each timepoint. Values shown are the mean ± 95% confidence intervals of pooled data from at least three separate experiments.

a Statistical significance (alpha = 0.05) for comparison to wild-type controls using Tukey-Kramer *post hoc* test.

Our finding that microtubules in *tub2-T178M* heterozygotes are longer and relatively stable compared to wild-type controls is surprising since our *in vitro* experiments indicate that purified tub2-T178M heterodimers exhibit loss of assembly activity (Figure 3). To determine whether the *tub2*-T178M acts as a recessive or dominant allele in cells, we compared *tub2*-T178M heterozygous cells to *tub2∆* heterozygous null cells that possess one copy of wild-type *TUB2* and the second copy is excised and replaced with a selectable marker (referred to as “*tub2∆*”; see *Materials and Methods*). If T178M acts as a recessive, loss of function mutant then it would be predicted to behave similarly to *tub2∆* heterozygous null. We find that *tub2∆* heterozygous null cells exhibit slower polymerization rates that are similar to T178M; but depolymerization rates in heterozygous null cells are significantly faster than T178M and dynamicity is significantly greater (Figures 4D,E; Table 1). Furthermore, microtubules in *tub2∆* heterozygous null cells exhibit a level of pause time that is similar to wild-type controls, and significantly less than that observed in *tub2*-T178M heterozygotes (Table 1). We conclude that the changes in microtubule dynamics seen in *tub2-*T178M heterozygous cells represents a dominant phenotype.

As a second test, we compared the sensitivity of heterozygous *tub2* mutants to drugs that bind to soluble tubulin and disrupt microtubule dynamics—benomyl and nocodazole (Kilmartin, 1981; Vasquez et al., 1997). We find that *tub2*-T178M heterozygotes are inhibited by low levels of benomyl (5 µg/ml) where *tub2∆* heterozygous null mutants grow similarly to wild-type controls (Figure 4F). This is consistent with *tub2*-T178M acting as a dominant negative. We also compared the benomyl sensitivity of cells heterozygous for *tub2*-C354S, a mutation substituting cysteine 354 for serine. Cysteine 354 is located far from the exchangeable GTP binding site, but the *tub2*-C354S mutation blocks the conformational changes in β-tubulin that normally accompany GTP hydrolysis (Geyer et al., 2015). The *tub2*-C354S mutation was previously shown to stabilize microtubules and exhibit long and persistently paused astral microtubules, reminiscent of *tub2*-T178M heterozygotes (Gupta et al., 2002). Despite the similar phenotypes for microtubule dynamics in cells, we find opposite phenotypes in the benomyl sensitivity assay—whereas *tub2*-T178M heterozygotes are highly sensitive to benomyl, *tub2*-C354S heterozygotes are strongly resistant (Figure 4F). We next tested how cell proliferation was affected by nocodazole. While *tub2*-T178M heterozygotes do not exhibit a proliferation phenotype in the absence of drug, upon the addition of low doses of nocodazole (5 µM) *tub2-*T178M mutants exhibit a significantly slower doubling time compared to wild-type controls and *tub2*∆ heterozygous null mutants (*p* < 0.01; Figure 4G). Interestingly, *tub2-*C354S mutants exhibit increased doubling time in the presence of 10 µM nocodazole compared to wild-type controls in 10 µM nocodazole (Figure 4G). Together, these results indicate that T178M exhibits a gain of function phenotype when expressed as a heterozygous allele, creating long and stable microtubules with exquisite sensitivity to the destabilizing drugs.

We considered two hypotheses to explain the hypersensitivity of *tub2*-T178M heterozygotes to benomyl and nocodazole: either the destabilizing drugs act in an additive fashion with the T178M mutation to suppress microtubule dynamics, or T178M causes underproduction of tubulin protein that leads to a higher proportion of drug-bound tubulin in the cells. To test the second hypothesis, we measured levels of total α- and β-tubulin in *tub2*-T178M heterozygotes and controls. We first used dilutions of purified yeast tubulin on western blots to create standard curves of signal per nanogram of α- or β-tubulin (Figure 4H). We then prepared lysates from wild-type, *tub2*-T178M heterozygous and *tub2*∆ heterozygous null cells under conditions to depolymerize microtubules and shift tubulin into the soluble fraction, and used western blots to estimate the number of α- and β-tubulin molecules per cell (see *Materials and Methods*; Figure 4I). Whereas *tub2*∆ heterozygous null cells show little change in the amount of α- or β-tubulin compared to wild-type controls, *tub2-*T178M heterozygotes exhibit significantly higher concentrations of both α- and β-tubulin (Figure 4J). We conclude that T178M does not cause underproduction of tubulin protein. Instead, T178M appears to cause an increase in the supply of α- and β-tubulin. Based on these results and our measurements of microtubule dynamics in *tub2*-T178M heterozygotes, we favor the hypothesis that the hypersensitivity of these mutants to benomyl and nocodazole reflects an additive suppression of microtubule dynamics.

### 2.6 Investigating the Mechanism of Kinetic Stabilization by T178M

Our findings that T178M mutant tubulin exhibits decreased assembly activity *in vitro* but creates stable microtubules in heterozygous cells is reminiscent of the effects of microtubule-targeting drugs that suppress tubulin on-off kinetics. For example, the drug vinblastine is thought to kinetically stabilize tubulin by increasing the affinity between heterodimers and eliminating the differences in assembly rate between GTP- and GDP-bound tubulin (Toso et al., 1993; Castle et al., 2017). We therefore explored how the T178M substitution might impact GTP-binding and/or hydrolysis activity to alter the nucleotide-dependence of tubulin assembly.

We considered two predictions based on structural studies of tubulin. The first prediction comes from a cryo-EM study comparing the conformational rearrangement of tubulin in different nucleotide states, which indicates a potentially state-specific role for threonine 178. When the GTP-mimicking analogue GMPCPP is bound at the exchangeable site, the hydroxyl group in the side chain of threonine 178 appears to hydrogen bond to the ribose of the guanosine nucleotide; but when GDP is bound, the T5 loop is repositioned and the hydrogen bond is lost (Manka and Moores, 2018). We reasoned that replacing threonine’s hydroxyl group with methionine’s S-methyl thioether could either weaken affinity for GTP and/or disrupt the ordering of the T5 loop exchangeable site when GTP is bound. An alternative prediction comes from crystal structures of tubulin oligomers mimicking the GTP-bound or GDP-bound states, which demonstrate a different role for the T5 loop (Nawrotek et al., 2011; Estevez-Gallego et al., 2020). Analysis of these structures indicates when GDP is bound at the exchangeable site, the T5 loop visits a “flipped in” conformation where threonine 178 hydrogen bonds to asparagine 101 in β-tubulin’s T3 loop. When GTP is bound at the exchangeable site, the T5 loop adopts a ‘flipped out’ conformation that allows asparagine 101 in the T3 loop to interact with α-tubulin across the longitudinal interface, and is thought to promote lateral interactions through an allosteric mechanism (Nawrotek et al., 2011). We reasoned that the T178M substitution could destabilize the “flipped in” conformation of T5 and thereby disrupt nucleotide-dependent regulation of interdimer interactions. Both of these predictions depend on the highly conserved hydroxyl group of the threonine 178 side chain.

To test whether the effects of the T178M mutation can be attributed to the loss of hydrogen bonding, we created an alternative substitution that lacks the hydroxyl group by introducing a valine substitution at position 178 of budding yeast *TUB2*. We predicted that the *tub2*-T178V mutant might phenocopy *tub2*-T178M in our assays of microtubule dynamics and drug sensitivity. However, our results show unique phenotypes for *tub2*-T178V. Astral microtubules in *tub2-*T178V heterozygous cells exhibit length distributions and dynamicity that are similar to microtubules in *tub2*-T178M heterozygotes, but with significantly faster rates of polymerization and depolymerization, and less time in pause (Figures 4D,E; Table 1). In addition, *tub2-*T178V cells exhibit different levels of sensitivity to benomyl and nocodazole. *tub2*-T178V heterozygotes show greater resistance to high concentrations of benomyl (15 µg/ml) than wild-type controls (Figure 4F) but are slightly more sensitive to high levels of nocodazole (10 µM) than wild-type controls (Figure 4G). Our results for *tub2*-T178V are reminiscent of the *tub2*-C354S mutant that constitutively mimics the GTP-bound state *tub2* (Figures 4F,G; Gupta et al., 2002; Geyer et al., 2015). We conclude that hydrogen bonding by threonine 178 is important for normal tubulin function and that loss of the threonine hydroxyl group may create a constitutive GTP-like state at the exchangeable site. However, the different phenotypes of T178M vs T178V mutants in our assays indicate that the introduction of the methionine side chain in T178M further disrupts the exchangeable site leading to slower polymerization and depolymerization and increased time in the paused state.

## 3 Discussion

We report two new tubulinopathy cases linked to *de novo*, heterozygous T178M missense mutations in either the *TUBB2A* or *TUBB3* β-tubulin isotypes. Comparing the structural features of brain development in these patients with a previously reported hypomyelinating leukodystrophy linked to an analogous T178M mutation in the *TUBB4A* isotype suggests specific developmental requirements for each β-tubulin isotype. In addition, we find that the T178M substitution creates a kinetically stabilized population of tubulin heterodimers that dominantly alters microtubule dynamics, representing a novel tubulinopathy mechanism. Together, our results underscore the importance of tubulin as the most abundant GTPase in the brain, and demonstrate how the family of tubulin isotypes may be used to meet shifting demands for tubulin activity at different times of development and in different cell types. The specific impacts of tubulinopathy mutations on brain development may therefore be related to both the timing and cell-type specific expression of the affected tubulin isotype and also the degree of molecular-level dysfunction in the mutant tubulin protein.

The MRI findings in both patients are consistent with other published cases of tubulin mutations in general and specifically in *TUBB2A* and *TUBB3* (Figure 1). These include the frequently reported findings of abnormal basal ganglia, thin corpus callosum, abnormal brainstem and vermis, and disorganized cerebral cortex referred to as dysgyria or tubulinopathy-associated dysgyria. All of these findings could reasonably be expected to occur due to defects in neuronal migration. While both patients had a similar array of malformations, patient #2’s MRI phenotype was overall more severe than patient #1. Patient #2 also presented distinct ocular motility phenotypes that suggest impairment of cranial nerves, consistent with previous reports linking ocular motility disorders to mutations in *TUBB3* (Tischfield et al., 2010).

A previous study reported a T178M mutation in *TUBB4A* in a patient with a milder hypomyelinating phenotype (Tonduti et al., 2016), although no MR images from the patient were published. In both of our patients, the cerebral white matter was low in volume but normally myelinated as is frequently the case in tubulinopathies. However, both of our patients had areas of abnormal white matter signal in the occipital regions. The appearance is non-specific, and while it could indicate a prior injury, given the association of tubulin mutations with hypomyelination, areas of focal hypomyelination are possible.

**FIGURE 5:**
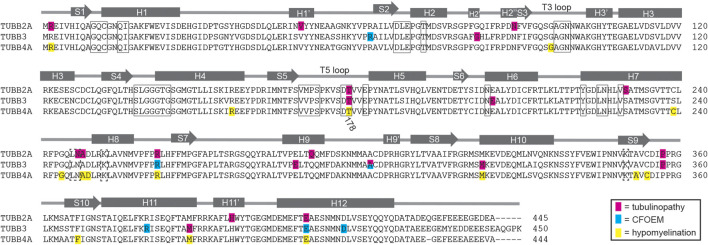
Diagram of identified patient mutations in TUBB2A, TUBB3 and TUBB4A. Amino acid sequence alignment of human TUBB2A, TUBB3 and TUBB4A overlayed with a model of β-tubulin domain structure adapted from Lowe et al. (2001). Residues mutated in tubulinopathy cases are highlighted in pink. Residues mutated in CFOEM3 cases are highlighted in blue. Residues mutated in hypomyelinating leukodystrophies are highlighted in yellow. Boxed residues contact nucleotide in the E-site, while dashed boxed residues contact nucleotide in the N-site.

In principle, tubulin isotypes provide metazoans with discrete transcriptional modules for meeting demands for tubulin subunits that shift during development and according to cell type. Our findings support the general conclusion that brain malformations caused by tubulinopathies are related to the timing and cell-type specific expression of the affected tubulin gene. The βII isotype is the most abundant β-tubulin in the adult mammalian brain (Banerjee et al., 1988). Our analysis shows that *TUBB2A*, one of the two genes for βII in humans, is expressed during brain development and maintained at high expression levels in neurons of the adult brain (Figure 2). Consistent with a role in brain development, a variety of *TUBB2A* mutations have been previously linked to cortical malformations (Figure 5; Cushion et al., 2014; Lee et al., 2014; Rodan et al., 2017; Ejaz et al., 2017; Brock et al., 2021). These patients commonly exhibit dysgyria, dysmorphic or hypoplastic corpus callosum and basal ganglia, and microcephaly; reminiscent of patient #1 (Figure 1). These findings are consistent with *TUBB2A* providing a major source of β-tubulin in neurons during brain development.

Although the *TUBB4A* isotype is also abundantly and specifically expressed in the mammalian brain, expression analysis and patient features indicate a role that is distinct from that of *TUBB2A* (Lewis et al., 1985). *TUBB4A* expression increases during postnatal brain development, and is strongly enriched in oligodendrocytes (Figure 2). Furthermore, it is well-established that mutations in *TUBB4A* are linked to hypomyelinating leukodystrophies, consistent with an important role for *TUBB4A* in oligodendrocyte function and the myelination of axons during postnatal brain development (Figure 5; Hersheson et al., 2013; Lohmann et al., 2013; Simons et al., 2013; Hamilton et al., 2014; Miyatake et al., 2014; Purnell et al., 2014; Lu et al., 2017). Thus the expression of *TUBB4A* in oligodendrocytes during postnatal brain development, or *TUBB2A* in neurons of the developing brain may provide an important mechanism for regulating tubulin activity in those cells; but it also creates specific vulnerabilities to isotype mutations.

The *TUBB3* isotype presents a more complicated story. TUBB3 is expressed in very few tissues of the human body. In the brain it is expressed in neurons, but not glia, and its expression is most abundant during fetal brain development and declines postnatally (Figure 2; Burgoyne et al., 1988; Chen et al., 2003; Katsetos et al., 2003). Importantly, *TUBB3* is expressed at much lower levels in neurons than other β-tubulin isotypes, including *TUBB2A* (Figure 2). Despite its low level of expression, mutations in *TUBB3* are linked to a variety of neuronal disorders, ranging from cortical malformations (Poirier et al., 2010; Oegema et al., 2015; Fukumura et al., 2016) to ocular motility disorders that arise from the failure of motor neurons to innervate eye muscles (Figure 5; Tischfield et al., 2010). Whereas the latter cases do not present obvious cortical malformations, patient #2 described in our study exhibits both cortical malformations and ocular motility disorders. This suggests that the developmental consequences of *TUBB3* missense mutations may be highly variable for different mutations. One possible explanation for this variability is that cortical neurons and motor neurons may require different levels of TUBB3 protein activity, and the impact of a particular *TUBB3* mutation may reflect the severity of its impact on protein function. Our understanding of the molecular impact of *TUBB3* mutations may therefore present a valuable window into unique functions of *TUBB3* that are important for neuronal development, despite its relatively low abundance.

Several models have been proposed to explain the dominance of tubulinopathy mutations. Fundamentally, the mutant tubulin protein produced by heterozygous tubulinopathy mutations represents no more than half of the tubulin pool in the cell; and in some cases, such as *TUBB3* tubulinopathies, may represent a much smaller fraction. In multiple reports, tubulinopathy mutations in α- or β-tubulin have been shown to disrupt the formation of stable heterodimers and lead to diminished tubulin protein levels in neurons and other cell expression models (Keays et al., 2007; Tian et al., 2010; Tischfield et al., 2010; Gartz Hanson et al., 2016; Buscaglia et al., 2020). This suggests that the cellular and developmental consequences of tubulinopathies can arise from haploinsufficiency of tubulin protein. Our results for the T178M mutation do not support this model. We find that the heterozygous expression of T178M β-tubulin in yeast cells leads to increased levels of α- and β-tubulin protein, compared to wild-type controls (Figure 4I). Importantly, the amount of soluble α- and β-tubulin protein measured in these experiments is increased, but the ratio of α- to β-tubulin is not obviously altered. It is therefore unlikely that the phenotypes of T178M mutant yeast cells are attributable to superstoichiometric levels of β-tubulin, which is known to be toxic in yeast cells (Burke et al., 1989; Weinstein and Solomon, 1990). The cause of the increased α- and β-tubulin in T178M heterozygous yeast is unclear, but could be related to the long and stable microtubules observed in mutant cells, which would be expected to deplete the cell’s soluble tubulin pool and might stimulate increased tubulin biogenesis through an autoregulatory mechanism that has been described in mammalian cells (Cleveland et al., 1981; Lin et al., 2020). Whether cells in T178M patients would also be expected to exhibit increased levels of α- and β-tubulin is unclear. Levels of α- and β-tubulin isotype proteins have not been measured in human cells, including the cells of the developing brain. If our mRNA analysis in Figure 2 is representative of protein levels, then TUBB2A protein, for example, could comprise approximately half of the β-tubulin in neurons. Therefore a heterozygous *TUBB2A* T178M mutant allele could supply approximately one quarter of the β-tubulin in neurons. But this is purely speculation, and it is important to note that mRNA levels are often not representative of protein levels and whether this is the case for tubulin has not been established. Our yeast experiments are designed to assess the impact of the T178M mutant in a system where the mutant allele is under endogenous expression and avoids artifacts generated by ectopic expression. Going forward, it will be important for the tubulinopathy field to determine isotype composition at the protein level for the cells of the developing brain, and then create experimental models that faithfully recapitulate this composition to test how shifts in the isotype blend may contribute to disease.

Our results present a possible alternative model in which the mutant tubulin acts in trans to dominantly suppress microtubule dynamics. Although purified T178M tubulin exhibits weakened assembly activity, it can co-assemble with wild-type tubulin in yeast cells to form microtubules that assemble and disassemble slowly, and dwell in a paused state with a stable polymer length (Figures 4B–E; Table 1). Microtubule dynamics vary across species and across cell types within a species, so it is important to consider that the magnitude of the effect of T178M on microtubule dynamics and related physiological consequences may be different in the human brain, where microtubules are regulated by a host of extrinsic factors. Going forward it may be informative to conduct further *in vitro* reconstitution experiments to measure microtubule dynamics when T178M mutant tubulin is blended at different proportions with wild-type tubulin. These experiments would provide direct evidence that T178M can co-assemble with wild-type tubulin, even in the absence of extrinsic microtubule regulators, and demonstrate how much T178M must be present in the lattice to exert effects on microtubule dynamics.

The attenuation of microtubule dynamics by T178M is reminiscent of the effects of microtubule-targeting drugs, and exemplified by vinblastine. Whereas adding vinblastine to soluble tubulin inhibits microtubule assembly, adding substoichiometric concentrations of vinblastine to pre-formed microtubules suppresses plus-end growth and shortening, leading to paused microtubules (Wilson et al., 1982; Toso et al., 1993; Castle et al., 2017). This apparent contradiction of inhibiting tubulin assembly and inhibiting microtubule disassembly is explained by vinblastine stabilizing conformations of tubulin at the microtubule ends that are not compatible with microtubule dynamics. Indeed, vinblastine binds near the longitudinal interface between tubulin heterodimers and stabilizes high-affinity tubulin-tubulin complexes that assemble into oligomeric spirals, quite distinct from the straight microtubule polymer (Na and Timasheff, 1982). Thus, even at low concentrations where only a fraction of tubulin is bound to drug (Toso et al., 1993), vinblastine appears to trap tubulin conformational states that potently suppress microtubule dynamics.

We propose that the T178M mutant may also create complexes at microtubule plus ends between mutant tubulins or between mutant and wild-type tubulins that suppress microtubule dynamics. Mechanistically, this is likely to involve breaking the network of conformational changes in β-tubulin’s T5 and T3 loops that normally accompany tubulin’s GTPase cycle and facilitate the free energy changes between nucleotide states that underlie dynamic instability (Nawrotek et al., 2011). T3 has been proposed to act as analogously to the Switch II region of canonical GTPases; it binds to the γ-phosphate of GTP and to the α-tubulin of the adjacent heterodimer, and is reordered after GTP hydrolysis to interact with T5 through hydrogen bonding between the T3 asparagine 101 and the T5 threonine 178 (Nogales et al., 1999; Nawrotek et al., 2011; Estevez-Gallego et al., 2020). Our results from heterozygous T178M or T178V mutants indicate that preventing this hydrogen bond in a portion of a cell’s tubulin pool creates hyperstable microtubules with suppressed dynamic instability (Figures 4B–E). Introduction of the methionine side chain in T178M appears to have consequences beyond loss of hydrogen bonding, since microtubules in T178M heterozygotes exhibit slower polymerization and depolymerization than those in T178V heterozygotes (Figure 4D; Table 1), and the cells are hypersensitive to benomyl (Figure 4F). These effects stand in contrast to the *TUBB3* E410K mutation that was identified in patients with ocular motility disorder and accelerates microtubule polymerization (Tischfield et al., 2010; Ti et al., 2018). To our knowledge, kinetic stabilization of microtubules by T178M represents a novel mechanism for tubulinopathies. Whether this model of kinetic stabilization is specific to the T178M mutation in β-tubulin or represents a mechanistic theme for other α and β tubulinopathy mutations will be the focus of future investigations.

## 4 Materials and Methods

### 4.1 Patient Sequencing

Whole exome sequencing and analysis of patient #1 was conducted by GeneDx (Gaithersberg, MD) using the Cortical Brain Malformations Panel, which analyzed the following genes: *ACTB*, *ACTG1*, *ADGRG1*, *AKT3*, *ARFGEF2*, *ARX*, *ASPM*, *ATP6V0A2*, *B3GALNT2*, *B3GNT1*, *CCND2*, *CUL4B*, *DCX*, *DYNC1H1*, *ERMARD*, *FAT4*, *FKRP*, *FKTN*, *FLNA*, *GMPPB*, *GPSM2*, *ISPD*, *KIF1BP*, *KIF2A*, *KIF5C*, *LAMB1*, *LAMC3*, *LARGE*, *NDE1*, *OCLN*, *LIS1*, *POMGNT1*, *POMGNT2*, *POMK*, *POMT1*, *POMT2*, *PQBP1*, *RAB18*, *RAB3GAP1*, *RAB3GAP2*, *RELN*, *RTTN*, *SRD5A3*, *SRPX2*, *TBC1D20*, *TMEM5*, *TUBA1A*, *TUBA8*, *TUBB*, *TUBB2A*, *TUBB2B*, *TUBB3*, *TUBB4A*, *TUBG1*, *VLDLR*, *WDR62*. Data are available in the ClinVar archive under accession SCV000321987.

Initial Next Generation Sequencing of patient #2 was conducted by Athena Diagnostics (Marlburough, MA) using a Congenital Muscular Dystrophy Advanced Sequencing Evaluation panel, which analyzed the following genes: *B3GALNT2*, *B3GNT1*, *CHKB*, *COL6A1*, *COL6A2*, *COL6A3*, *DNM2*, *DPM2*, *FHL1*, *FKRP*, *FKTN*, *ISPD*, *ITGA7*, *LAMA2*, *LARGE*, *LMNA*, *POMGNT1*, *POMGNT2*, *POMT1*, *POMT2*, *SEPN1*, *TCAP*, *TMEM5*. Subsequently, genome sequencing analysis was conducted by GeneDx using the Cortical Brain Malformations Panel described above.

### 4.2 RNA Transcript Expression Data

RNA-sequencing data comparing RNA transcript expression across pre- and post-natal developmental time were obtained from both male and female cortical and subcortical samples published in the developmental transcriptome on brainspan.org (Miller et al., 2014). Data for *TUBB4A*, *TUBB3*, and *TUBB2A* from 8 pcw to 40 years were binned into three categories representative of different phases of development. “Fetal” includes data from 8 to 37 pcw, “postnatal” includes 4 months to 11 years, and “adult” includes 13–40 years.

RNA transcript expression data of the nine human β-tubulin isotypes were obtained from brainrnaseq.org (Zhang et al., 2016). Data were collected from adult samples of whole cortex, neurons, oligodendrocytes, and astrocytes. Fetal astrocyte samples were also obtained.

### 4.3 *In Vitro* Tubulin Assembly Assays

Wild-type or T178M mutant tubulin was purified using the protocol developed by Johnson and colleagues (Johnson et al., 2011). The T178M mutation was introduced using site-directed mutagenesis (Agilent Technologies; Santa Clara, CA) into a plasmid-bound copy of budding yeast *TUB2* under the control of a galactose inducible promoter. Purified tubulin was incubated with fragments of sea urchin axonemes at 30°C and imaged by Differential Interference Contrast microscopy to measure microtubule dynamics. Image series of individual microtubules were converted into kymographs, and rates were determined as the change in length divided by the change in time for each polymerization event.

### 4.4 Yeast Strains and Manipulation

General yeast manipulation, media and transformation were performed by standard methods. A list of yeast strains is provided in Supplementary Table S1. To construct T178M and T178V mutations, we used site-directed mutagenesis (Agilent Technologies; Santa Clara, CA) of a plasmid containing the *TUB2* locus, from 450 base pairs 5’ of the coding sequence through 427 base pairs 3’ of the coding sequence. Mutations were confirmed by sequencing, and then the mutant *TUB2* plus a *TRP1* selectable marker was amplified from the plasmid by PCR, and integrated into the native *TUB2* genomic locus. The integrated mutants were confirmed by sequencing. The Bik1-3GFP integrating plasmid was a gift from Dr. David Pellman (Harvard University).

### 4.5 *In Vivo* Microtubule Dynamics Assays

Asynchronous cultures of cell expressing Bik1-3GFP were grown to early log phase in nonfluorescent medium and mounted in imaging chambers (Fees et al., 2017). Images were collected on a Nikon Ti-E microscope equipped with a 1.45 NA 100× CFI Plan Apo objective, piezo electric stage (Physik Instrumente, Auburn, MA), spinning disk confocal scanner unit (CSU10; Yokogawa), 488 and 561-nm lasers (Agilent Technologies, Santa Clara, CA), and an EMCCD camera (iXon Ultra 897; Andor Technology, Belfast, United Kingdom) using NIS Elements software (Nikon). During imaging, the temperature of the stage was maintained at 30°C.

Microtubule dynamics were analyzed by measuring the distance between Bik1-3GFP foci at astral microtubule plus ends and spindle poles at 5 s intervals for 8 min. This analysis was conducted in pre-anaphase cells, which typically exhibit one or two astral microtubules emanating from each spindle pole. For these experiments, the sample sizes were as follows: +/+, *n* = 27 cells; ∆/+, *n* = 30 cells; T178M/+, *n* = 28 cells; T178V/+, *n* = 18 cells. Assembly and disassembly events were defined as at least three contiguous data points that produced length change greater than 500 nm and a coefficient of variation ≥0.80. Pause events were defined as data points that did not meet the criteria for assembly or disassembly, and did not show significant length change. Catastrophes were defined as transitions from assembly or pause to disassembly. Catastrophe frequencies were determined for individual astral microtubules by dividing the number of catastrophe events by the total time spent in assembly and pause states. Rescues were defined as transitions from disassembly or pause to assembly. Rescue frequencies were determined for individual astral microtubules by dividing the number of rescue events by the total time disassembly and pause states. Microtubule dynamicity was calculated by the total change in length (growing and shrinking) divided by the change in time and expressed in tubulin subunits changed per second (Toso et al., 1993).

### 4.6 Benomyl Sensitivity

Cells with were grown to saturation at 30°C in rich media and a 10-fold dilution series of each was spotted to either YPD or YPD supplemented with 5 or 15 µg/ml benomyl. Plates were incubated at 30°C for 2–3 days before imaging. The full experiment was completed at least three times for each genotype.

### 4.7 Liquid Growth Assay

Cells were grown to saturation at 30°C in rich media and diluted 50-fold into fresh rich media. One hundred and ninety-eight microlitres of each diluted culture was then aliquoted into the wells of a 96-well plate, with three replicates of each condition per experiment. Nocodazole in DMSO was diluted 1:100 into each well to maintain 1% DMSO. The plate was incubated at 30°C with single orbital shaking in an Epoch 2 plate reader (BioTek; Winooski, VT). The OD_600_ was measured every 5 min for 24 h. Doubling time was calculated by fitting the growth curves to a nonlinear exponential growth curve as previously published (Fees et al., 2018). Data points plotted as dots represent doubling time measurements for each technical replicate from three separate experiments, and the mean value of the technical replicates within each experiment is represented as a triangle. ANOVA with a Tukey *post-hoc* test was completed on the means of the technical replicates using Prism 9 (GraphPad Software; San Diego, CA).

## 4.8 Quantitative Western Blot

Tubulin protein abundance per cell was determined by western blot of whole yeast cell extract (Zhang et al., 2011). Wild-type, heterozygous null, or mutant tubulin cells were grown to log phase at 30°C in 50 ml of rich media, then moved to a nutator at 4°C for 1 h to depolymerize microtubules. Prior to lysate preparation, cell density was determined by hemocytometer counts. For each culture in each experiment, approximately 5 × 10^7^ cells were pelleted at 4°C. Cell pellets were resuspended in 2 M lithium acetate and incubated on ice for 5 min, then pelleted and resuspended in 400 mM NaOH for another 5 min on ice to permeabilize the cell wall. Permeabilized cells were pelleted and resuspended in 50 µl of 2.5× Laemmli buffer, boiled for 5 min for complete cell lysis, and centrifuged before loading onto the gel. Standards of purified yeast tubulin were prepared by diluting protein to 2.5 ng/µl in 2.5× Laemmli buffer. Dilution series of purified protein (4, 10, 15, 30, and 40 ng) and lysate samples corresponding to 3.5, 4.5, 6, and 8 × 10^6^ cells and were run on a 10% Bis-Tris PAGE, transferred to PVDF and blocked for 1 h at room temperature. Membranes were probed with mouse-anti-α-tubulin (4A1; at 1:100; Piperno and Fuller, 1985), mouse-anti-β-tubulin (E7; at 1:100; Developmental Studies Hybridoma Bank, University of Iowa), rabbi-anti-Zwf1 (Glucose-6-phosphate dehydrogenase; Sigma A9521; at 1:10,000) and followed by goat-anti-mouse-680 (LI-COR 926-68070, Superior, NE; at 1:15,000) and goat-anti-rabbit-800 (LI-COR 926-32211; at 1:15,000) secondary antibodies and imaged on an Odyssey Imager (LI-COR Biosciences). Band intensities were quantified using the gel analysis plug-in in FIJI.

To calculate the number of tubulin molecules per cell, a standard curve of signal per nanogram of tubulin was first calculated for the purified proteins. Nanograms was converted to molecules of α- or β-tubulin by:

$$ng*\frac{1\;Da}{1.66*10^{-15}ng}*\frac{1\;kDa}{1000\;Da}*\frac{1\;molecule\;}{MW\;kDa}*\frac{1}{cells\;loaded}=\frac{molecules}{cell}$$

## Data Availability

The data presented in the study are deposited in the ClinVar repository, accession number SCV000321987.
