# Supplementary material for: Kinetically Stabilizing Mutations in Beta Tubulins Create Isotype-Specific Brain Malformations
Source: Front Cell Dev Biol. 2021 Nov 18;9:765992. doi: 10.3389/fcell.2021.765992 (PMC8637541; doi:10.3389/fcell.2021.765992)
Supplement: Supplementary file 1 [file DataSheet1.PDF]

Table S1. Yeast strains used in this study

Strain #	Genotype	source
yJM0091/ YEF473	*MAT**a**/α ura3-52/ura3-52 lys2-801/lys2-801 leu2-Δ1/leu2-Δ1 his3-Δ200/his3-Δ200 trp1-Δ63/trp1-Δ63*	(Bi and Pringle, 1996)
yJM0309	*MAT**a**/α BIK1-3GFP::TRP/BIK1 ura3-52/ura3-52 lys2-801/lys2-801 leu2-Δ1/leu2-Δ1 his3-Δ200/his3-Δ200 trp1-Δ63 /trp1-Δ63*	(Aiken et al., 2014)
yJM0592	*MAT**a**/α tub2::HIS3MX6/TUB2 ura3-52/ura3-52 lys2-801/lys2-801 leu2-Δ1/leu2-Δ1 his3-Δ200/his3-Δ200 trp1-Δ63/trp1-Δ63*	This study
yJM1209	*MAT**a**/α tub2-C354S+331::TRP1/TUB2 ura3-52/ura3-52 lys2-801/lys2-801 leu2-Δ1/leu2-Δ1 his3-Δ200/his3-Δ200 trp1-Δ63 /trp1-Δ63*	(Estrem et al., 2017)
yJM2065	*MAT**a**/α tub2::CaURA3/TUB2 ura3-52/ura3-52 lys2-801/lys2-801 leu2-Δ1/leu2-Δ1 his3-Δ200/his3-Δ200 trp1-Δ63/trp1-Δ63*	This study
yJM3044	*MAT**a**/α tub2-T178M+331::TRP1/TUB2 ura3-52/ura3-52 lys2-801/lys2-801 leu2-Δ1/leu2-Δ1 his3-Δ200/his3-Δ200 trp1-Δ63/trp1-Δ63*	This study
yJM3045	*MAT**a**/α tub2-T178M+331::TRP1/TUB2 ura3-52/ura3-52 lys2-801/lys2-801 leu2-Δ1/leu2-Δ1 his3-Δ200/his3-Δ200 trp1-Δ63/trp1-Δ63*	This study
yJM3171	*MAT**a**/α tub2-T178V+331::TRP1/TUB2 ura3-52/ura3-52 lys2-801/lys2-801 leu2-Δ1/leu2-Δ1 his3-Δ200/his3-Δ200 trp1-Δ63/trp1-Δ63*	This study
yJM3335	*MAT**a**/α Bik1-3GFP::TRP/BIK1 tub2-T178M+331::HIS/TUB2 ura3-52/ura3-52 lys2-801/lys2-801 leu2-Δ1/leu2-Δ1 his3-Δ200/his3-Δ200 trp1-Δ63 /trp1-Δ63*	This study
yJM3339	*MAT**a**/α tub2-T178V+331::HIS3/TUB2 ura3-52/ura3-52 lys2-801/lys2-801 leu2-Δ1/leu2-Δ1 his3-Δ200/his3-Δ200 trp1-Δ63/trp1-Δ63*	This study
yJM3340	*MAT**a**/α BIK1-3GFP::TRP/BIK1 tub2-T178V+331::HIS/TUB2 ura3-52/ura3-52 lys2-801/lys2-801 leu2-Δ1/leu2-Δ1 his3-Δ200/his3-Δ200 trp1-Δ63 /trp1-Δ63*	This study
yJM3337	*MAT**a**/α Bik1-3GFP::TRP/BIK1 tub2Δ::CaURA3/TUB2 ura3-52/ura3-52 lys2-801/lys2-801 leu2-Δ1/leu2-Δ1 his3-Δ200/his3-Δ200 trp1-Δ63 /trp1-Δ63*	This study
yJM3415	*MAT**a**/α BIK1-3GFP::TRP/BIK1 ura3-52/ura3-52 lys2-801/lys2-801 leu2-Δ1/leu2-Δ1 his3-Δ200/his3-Δ200 trp1-Δ63 /trp1-Δ63*	This study
yJM3416	*MAT**a**/α BIK1-3GFP::TRP/BIK1 tub2-T178M+331::HIS/TUB2 ura3-52/ura3-52 lys2-801/lys2-801 leu2-Δ1/leu2-Δ1 his3-Δ200/his3-Δ200 trp1-Δ63 /trp1-Δ63*	This study
yJM3417	*MAT**a**/α Bik1-3GFP::TRP/BIK1 tub2Δ::CaURA3/TUB2 ura3-52/ura3-52 lys2-801/lys2-801 leu2-Δ1/leu2-Δ1 his3-Δ200/his3-Δ200 trp1-Δ63 /trp1-Δ63*	This study
yJM3613	*MAT**a**/α ura3-52/ura3-52 lys2-801/lys2-801 leu2-Δ1/leu2-Δ1 his3-Δ200/his3-Δ200 trp1-Δ63/trp1-Δ63*	This study
yJM3614	*MAT**a**/α ura3-52/ura3-52 lys2-801/lys2-801 leu2-Δ1/leu2-Δ1 his3-Δ200/his3-Δ200 trp1-Δ63/trp1-Δ63*	This study

REFERENCES

Aiken, J., Sept, D., Costanzo, M., Boone, C., Cooper, J.A., and Moore, J.K. (2014). Genome-wide analysis reveals novel and discrete functions for tubulin carboxy-terminal tails. Curr Biol. 24**,** 1295-1303.

Bi, E. and Pringle, J. (1996). ZDS1 and ZDS2, genes whose products may regulate Cdc42p in Saccharomyces cerevisiae. MCB. 16, 5264-5275.

Estrem, C., Fees, C.P., and Moore, J.K. (2017). Dynein is regulated by the stability of its microtubule track. J Cell Biol. 216**,** 2047-2058.
